# Supplementary material for: Nipah virus W protein harnesses nuclear 14-3-3 to inhibit NF-κB-induced proinflammatory response
Source: Commun Biol. 2021 Nov 16;4:1292. doi: 10.1038/s42003-021-02797-5 (PMC8595879; doi:10.1038/s42003-021-02797-5)
Supplement: Supplementary file 2 — Supplementary Information [file 42003_2021_2797_MOESM2_ESM.pdf]

SUPPLEMENTARY MATERIEL

Nipah virus W protein harnesses nuclear 14-3-3 to inhibit NF-κB-induced proinflammatory response

François Enchéry[1,#], Claire Dumont[1,#], Mathieu Iampietro[1,#], Rodolphe Pelissier[1,#], Noémie Aurine[1], Louis-Marie Bloyet[1], Caroline Carbonnelle[2], Cyrille Mathieu[1], Chloé Journo[1], Denis Gerlier[1,#] and Branka Horvat[1*]

Authors' affiliations:

[1]CIRI, Centre International de Recherche en Infectiologie, Inserm U1111, CNRS, UMR5308, Univ Lyon, Université Claude Bernard Lyon 1, École Normale Supérieure de Lyon, France

[2]INSERM- Laboratoire P4 Jean Mérieux-21 Avenue Tony Garnier, 69365 Lyon, France

[3]These authors contributed equally

Content:

Supplementary Figure 1 a, Comparison of W sequences of NiV Bangladesh (NiV-B, AY988601), NiV Malaysia (NiV-M, AF212302) and Hendra virus (HeV MN062017) isolates; b, Relative expression level of viral proteins.

Supplementary Figure 2. a, Cell viability (left panel) and transfection efficiency (right panel) in the ImageStreamX analysis presented in Figure 2b,c; b, c Expression of FLAG-tagged W protein constructs used throughout Figures 2-6 and Supplemental Figures 2-8.

Supplementary Figure 3. W-CTD-S449 is required for the inhibition of NF-κB activation but dispensable for the nuclear accumulation of W.

Supplementary Figure 4. Phosphorylation of S449 is required for W protein interaction with 14-3-3.

Supplementary Figure 5. W-CTD S449 is required for the inhibition of the phosphorylation of NF-kB p65.

Supplementary Figure 6. W protein induces 14-3-3 nuclear accumulation in non-stimulated cells.

Supplementary Figure 7. W-CTD protein induces 14-3-3 nuclear accumulation.

Supplementary Figure 8. W-NLS is responsible for the nuclear localization of 14-3-3.

Supplementary Table 1. Statistical analysis of cytokine production results presented in Figure 1b.

Supplementary Table 2. Summary of available experimental data on the ability of NiV W and its variants to accumulate in the nucleus, to bind to 14-3-3, to alter NF-kB p65 nucleo-cytoplasmic distribution and inhibit NF- kB -mediated signalling.

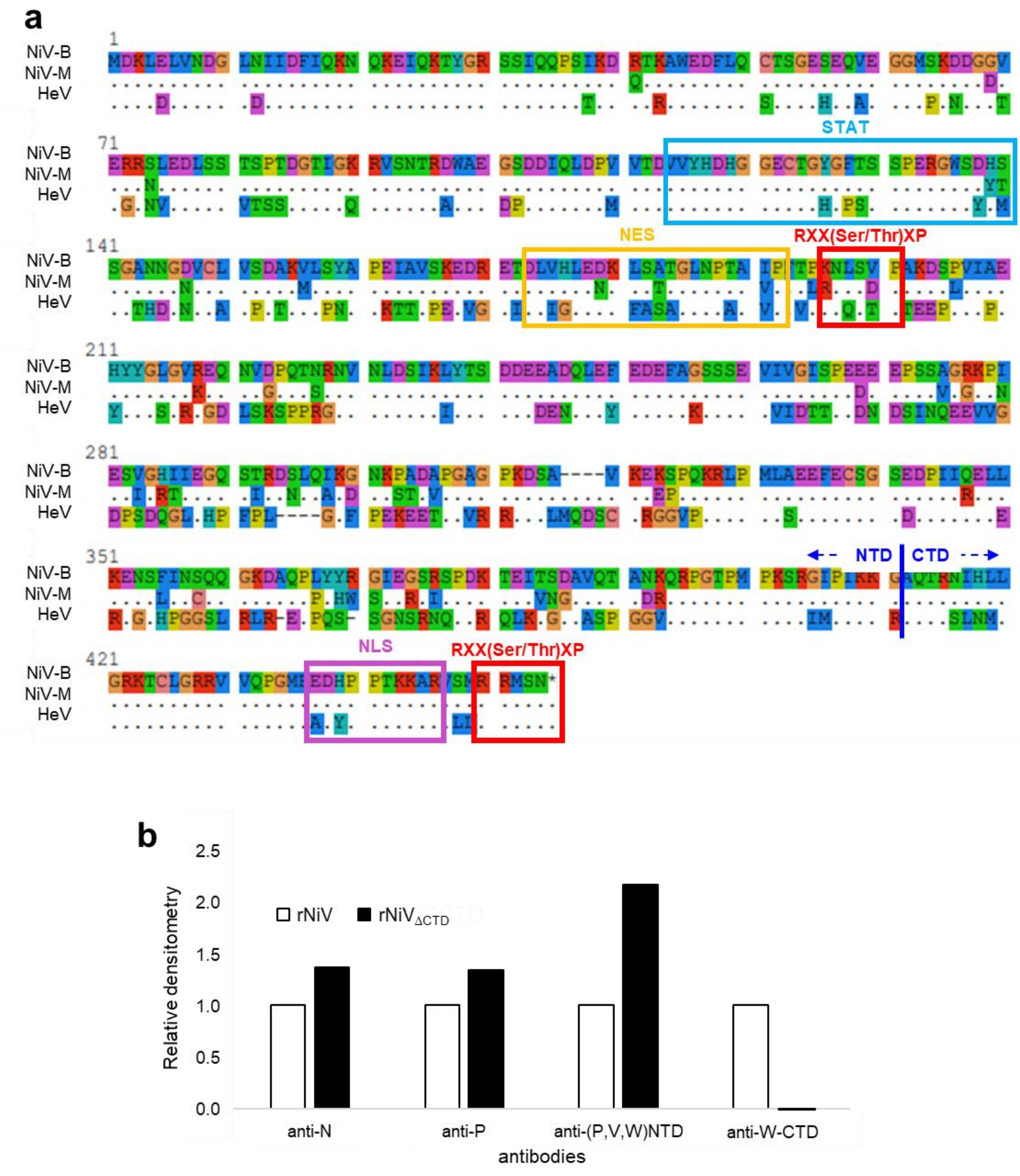

Supplementary Figure 1a, Comparison of W sequences of NiV Bangladesh (NiV-B, AY988601), NiV Malaysia (NiV-M, AF212302) and Hendra virus (HeV MN062017) isolates. W-NTD and CTD of the two NiV strains share 87% and 100% identity respectively. W-NTD of HeV shares with NiV-B and NiV-M 56% and 57% identity, respectively. W-CTD of HeV share with NiV (M and B) 81% identity. STAT1/ 4-binding domain, NES and NLS are indicated by squares. Red squares correspond to the RXX(Ser/Thr)XP motif presenting a potential site of interaction with 14-3-3.

b, Relative expression level of viral proteins as assessed by densitometry of western blot analysis of cells infected either by rNiV or rNiV$_{\Delta CTD}$ shown in **Figure 1d**. Quantification of each protein expressed by the two recombinant viruses was performed by normalizing values obtained by the mean value of GAPDH from all conditions. Then, the fold expression of viral proteins expressed by rNiV$_{\Delta CTD}$ compared to rNiV was determined using the ratio rNiV$_{\Delta CTD}$/rNiV (rNiV-related protein fold expression corresponding to 1).

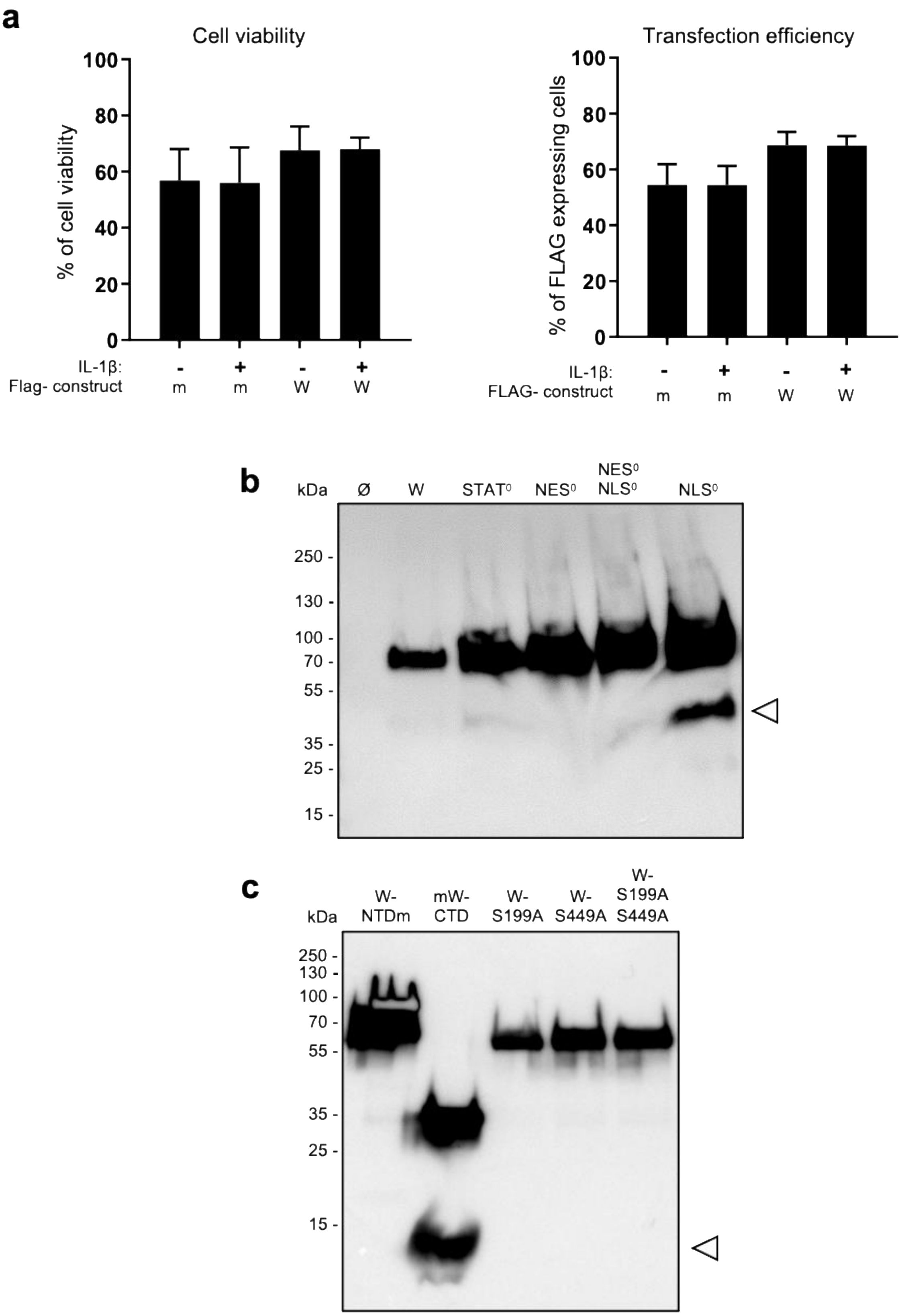

Supplementary Figure 2.

a, Cell viability (left panel) and transfection efficiency (right panel) in the ImageStreamX analysis presented in Figure 2b, c**.** HeLa cells were transfected with plasmids encoding either FLAG-mCherry (m) or FLAG-W (W) protein and stimulated or not with 10 ng/ml of IL-1β for 20 min. Cells were first labelled with eF660 dye to stain dead cells, then fixed and immunostained with an anti-FLAG antibody. The proportion of live (eF660 negative) and transfected cells was quantified using ImageStreamX. The data represent mean values of 3 independent experiments showed as mean ± SD **b, c Expression of FLAG-tagged W protein constructs used throughout Figures 2-6 and Supplemental Figures 2-8**. HeLa cells were transfected with plasmids encoding Flag-tagged W or truncated/ variant proteins, or an empty vector (Ø) as control. 24h post transfection, cells were lysed and FLAG-tagged proteins were revealed using mouse α-FLAG antibodies. Major protein degradation products are indicated by white arrows.

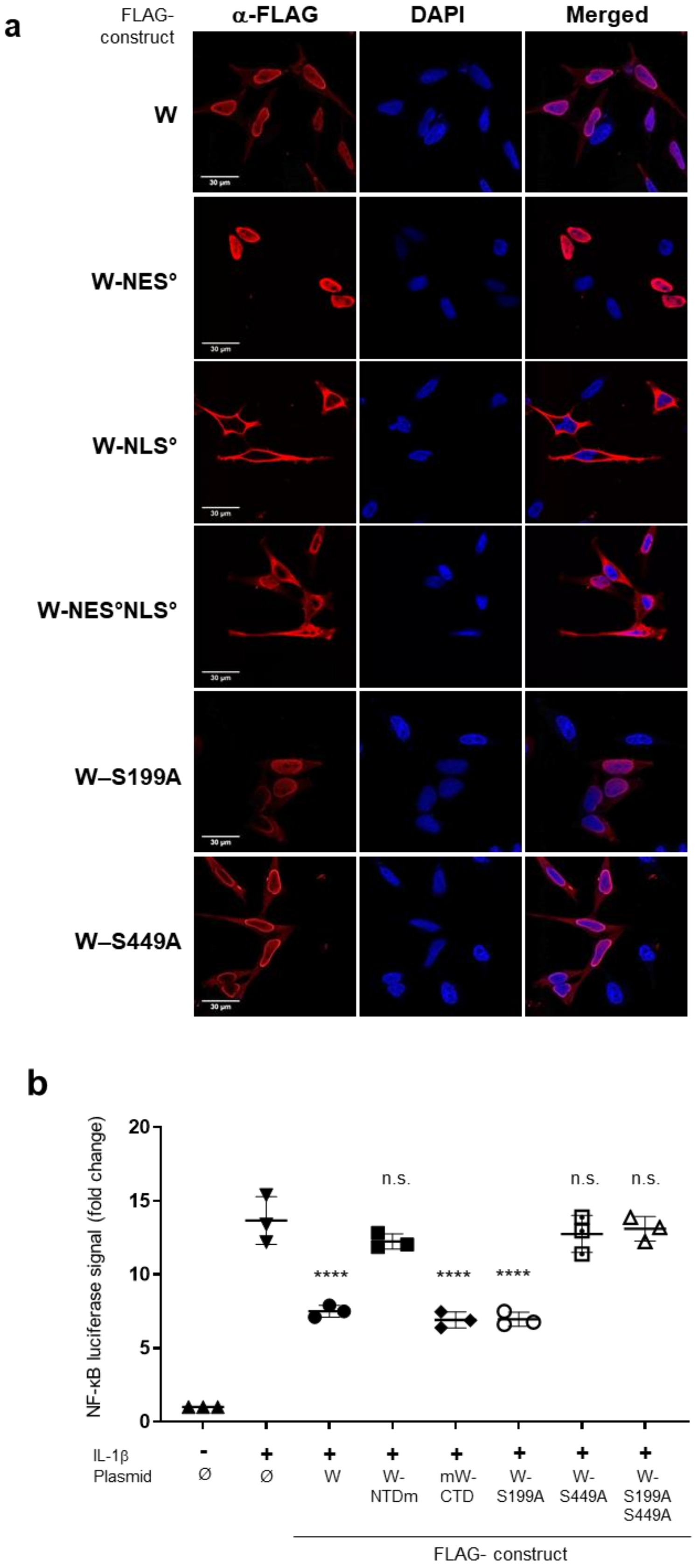

Supplementary Figure 3. W-CTD-S449 is required for the inhibition of NF-κB activation but dispensable for the nuclear accumulation of W. (**a**) HeLa cells were transfected with plasmids encoding FLAG-tagged W protein variants. 20 h later, cells were fixed, permeabilized, stained with DAPI and mouse α-FLAG antibody and analyzed by confocal microscopy. (**b**) HeLa cells were transfected with a plasmid encoding NF-κB_luc and plasmids encoding FLAG-tagged W protein variants. Transfection with Renilla luciferase was used for the normalization of obtained results. 20 h later, cells were stimulated with 10 ng/ml of IL-1β for 4 h before measurement of NF-κB activity by luminescence quantification. Data are presented as mean values of at least 3 independent experiments with each point done in triplicate ± SD. Data obtained with W proteins were compared with the signal observed after IL-1 stimulation in cells transfected with the empty vector (**** p<0.0001, n.s. not significant, using ordinary One-way ANOVA followed by a Tukey's multiple comparisons test).

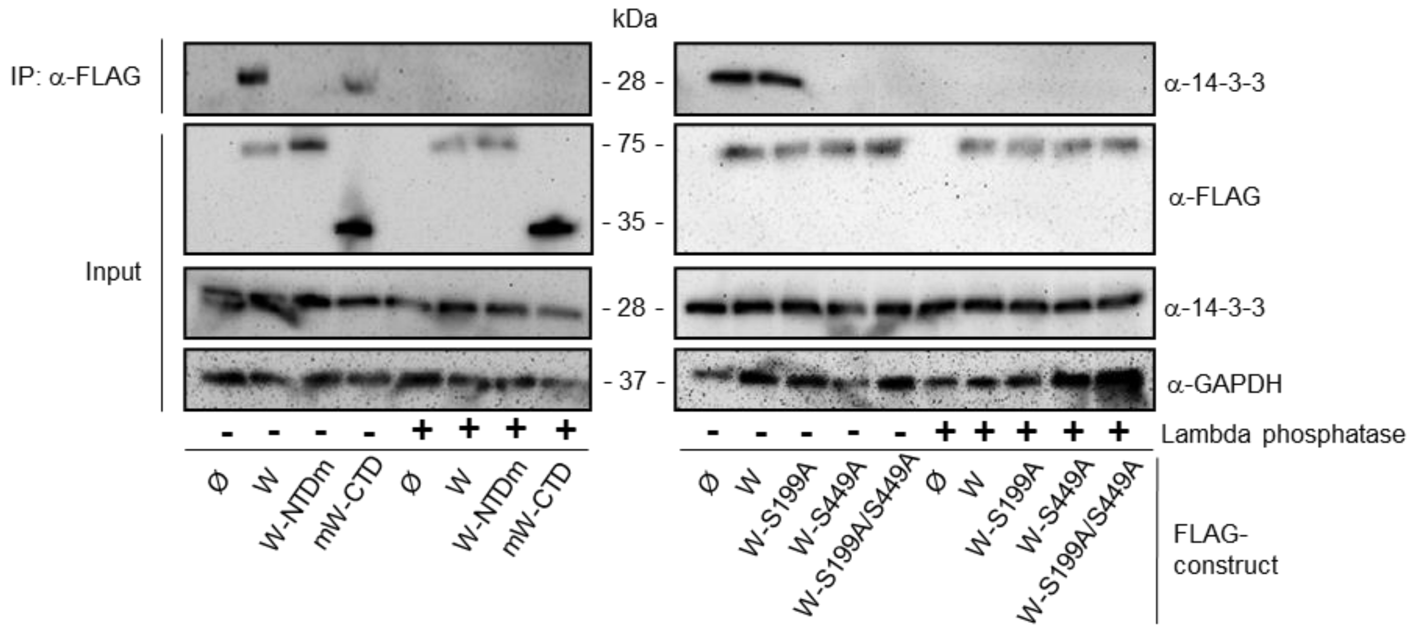

Supplementary Figure 4. Phosphorylation of S449 is required for W protein interaction with 14-3-3. HPMEC cells were transfected with a plasmid encoding FLAG-tagged W or W truncated/variant proteins; an empty vector (Ø) was used as a control. Cellular extracts were treated or not with Lambda phosphatase prior to immunoprecipitation. Co-immunoprecipitation of exogenous FLAG-W proteins with endogenous 14-3-3 proteins using anti-FLAG antibodies bound to magnetic beads and detected by western blotting (IB) using rabbit anti-pan 14-3-3 antibodies. Input cell extracts were analyzed by western blotting using anti-FLAG, anti-14-3-3 and anti-GAPDH antibodies.

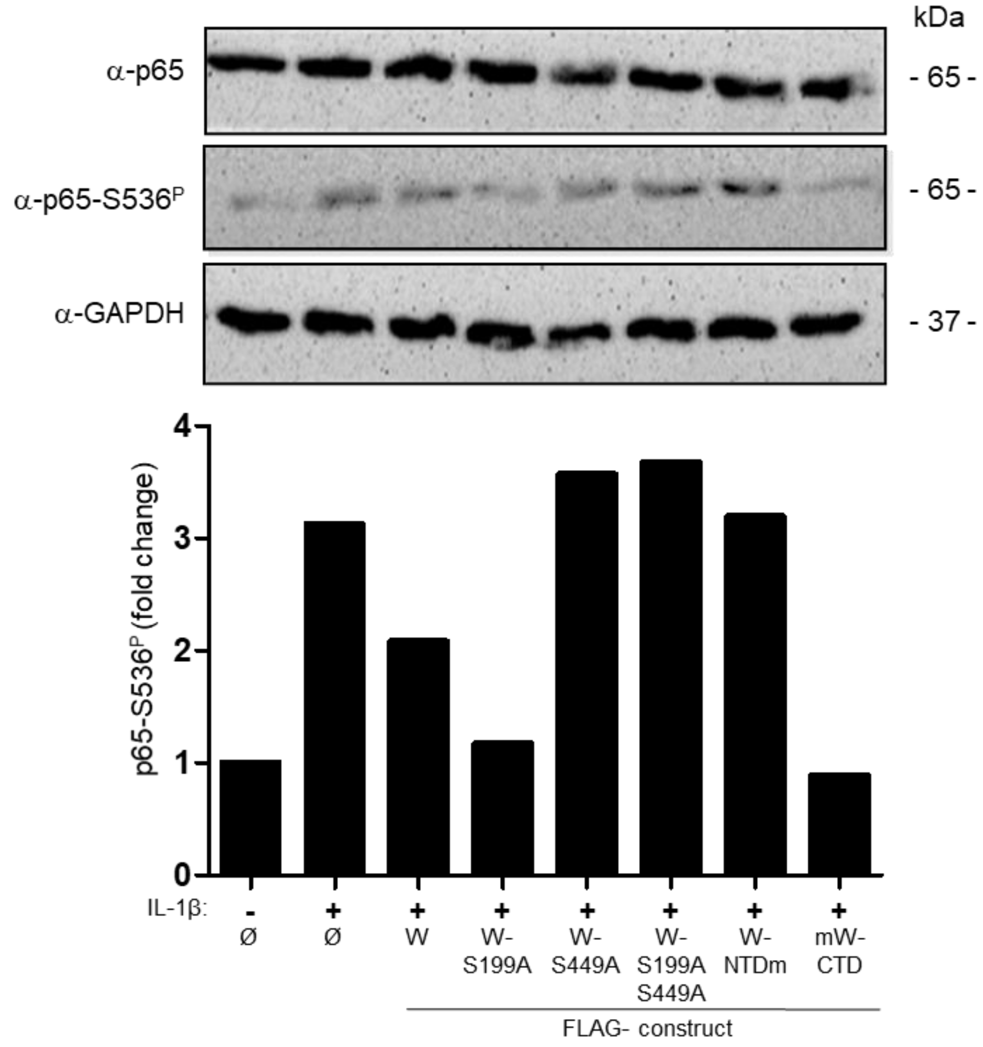

Supplementary Figure 5. W-CTD S449 is required for the inhibition of the phosphorylation of NF-kB p65. Western blot and densitometry measurements of one set of representative data from three independent experiments, the mean data of which are shown in Figure 4b (note the different order of W constructs), aiming at detecting p65-S536^{P} phosphorylation level.

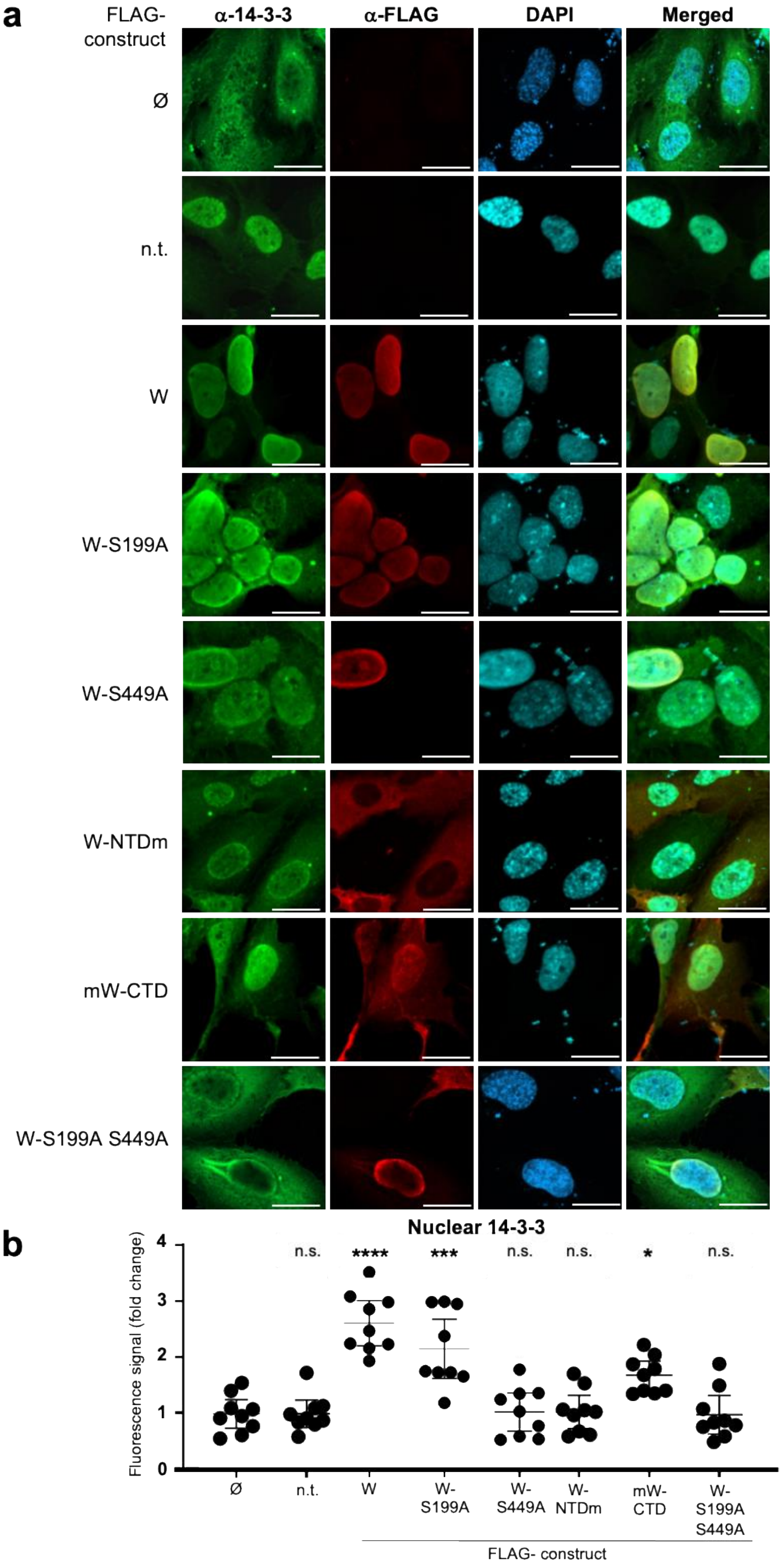

Supplementary Figure 6. W protein induces 14-3-3 nuclear accumulation in non-stimulated cells. a, HPMEC cells were either non-transfected (n.t.) or transfected with plasmids empty (Ø) or encoding FLAG-W constructs. Cells were fixed, permeabilized, stained with DAPI, a α-Flag mouse antibody and anti-14-3-3 rabbit antiserum and analysed by confocal microscopy. (Scale bar = 20µm). **b,** Fluorescence intensity of nuclear 14-3-3 protein was measured in 9 randomly chosen cells and expressed as the fold change of that of n.t. cells. Error bars represent the confidence interval of the mean (CI 95%) for 9 cells. Statistical significance was assessed using a nonparametric Kruskal-Wallis test with Dunn's multiple comparisons; * $p<0.05$, *** $p< 0.001$ and **** $p<0.0001$.

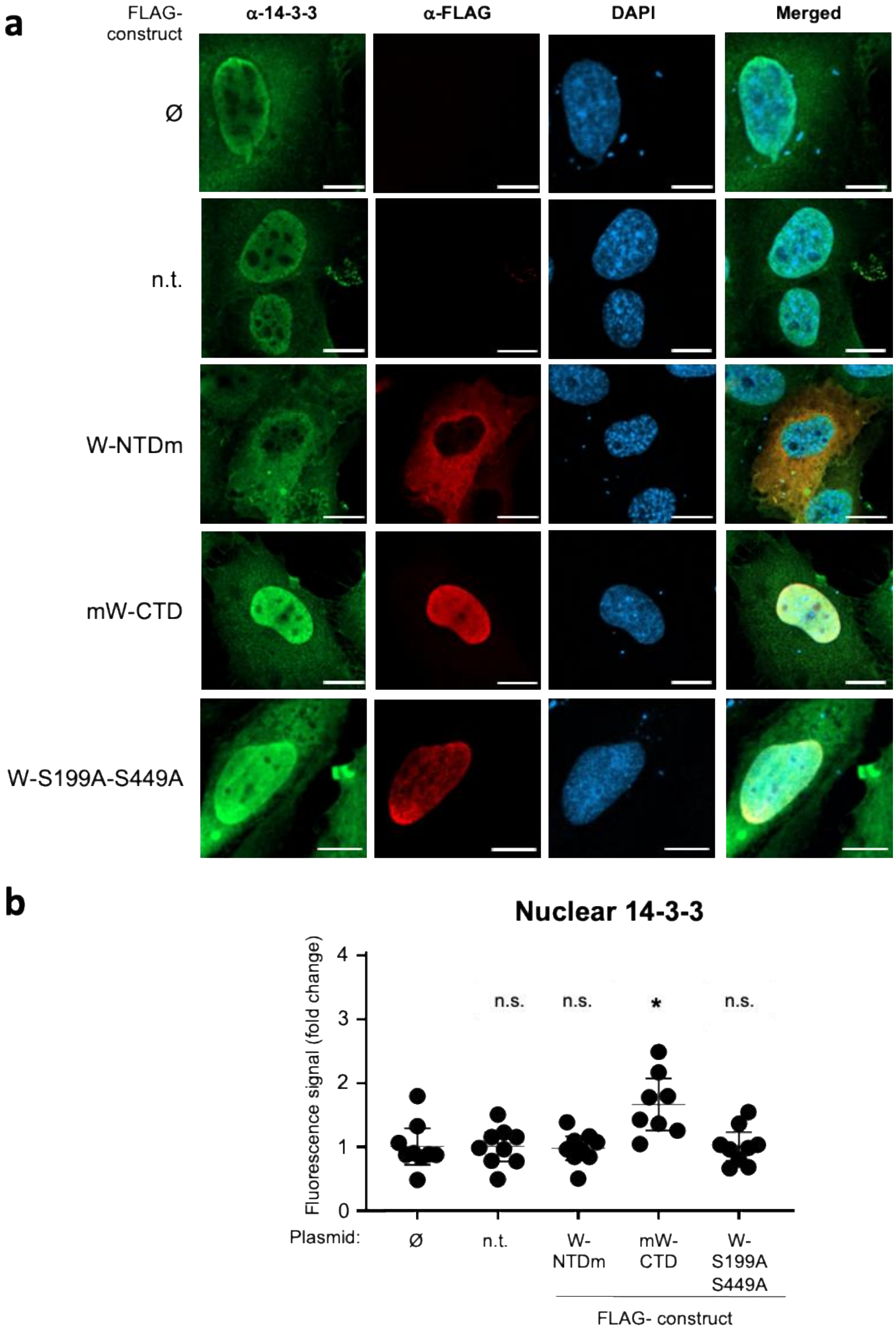

Supplementary Figure 7. W-CTD protein induces 14-3-3 nuclear accumulation. a, b, HPMEC cells were transfected with plasmids encoding FLAG-tagged W protein constructs, or an empty vector (Ø), or left non-transfected (n.t.). Cells were stimulated 20 h later with 10 ng/ml of IL-1β for 20 min before being fixed, permeabilized, stained with DAPI, an anti-FLAG mouse antibody and anti-14-3-3 rabbit antiserum and analyzed by confocal microscopy (scalebar = 10 µm). **b**, The fluorescence intensity of nuclear 14-3-3 protein was measured and expressed as fold change of the nuclear fluorescence signal of non-transfected IL-1β-stimulated cells. Error bars represent the confidence interval of the mean (CI 95%) for 9 cells. Each combination has been done in three different wells performed in two independent experiments. Statistical significance was assessed using a nonparametric Kruskal-Wallis test with Dunn's multiple comparisons; * $p<0.05$.

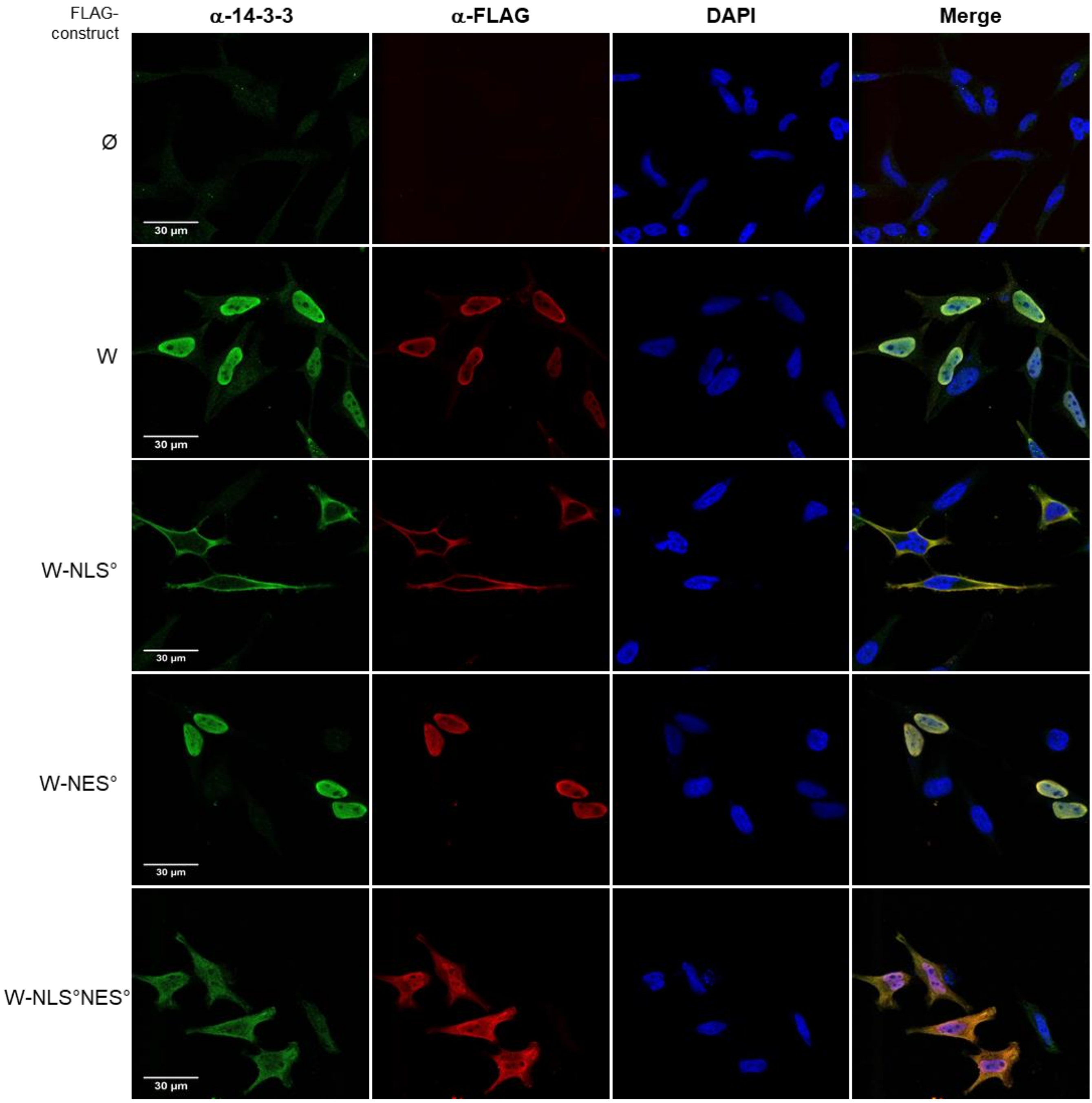

Supplementary Figure 8. W-NLS is responsible for the nuclear localization of 14-3-3. HeLa cells were transfected with plasmids encoding FLAG-tagged W protein variants or an empty vector (Ø) as a control. 20 h later, cells were fixed, permeabilized, stained with DAPI, a mouse anti-FLAG antibody and a pan anti-14-3-3 rabbit, and analyzed by confocal microscopy.

Supplementary Table 1.

Statistical analysis of cytokine production results presented in Figure 1b.

**Cytokine***	**Equation of the linear model**	**p-value**
IL-1β	$y = 0.038\,x + 0.80$	0.1185
IL-6	$y = 0.24\,x + 1.64$	0.2778
TNFα	$y = 2.15\,x + 7.72$	0.1404
MIP-1α	$y = 0.0044\,x + 8.75$	0.9755
MIP-1β	$y = 0.175\,x + 1.99$	0.0576
IL-12/23	$y = 0.49\,x + 10.2$	0.0515
IL-2	$y = 2.54\,x + 27$	0.0024
Perforin	$y = 1170\,x + 1743$	0.0014
IFNγ	$y = 11.6\,x - 13.4$	0.0031

*****Nipah virus-infected AGM (n=3) were analyzed for the presence of NF-κB-controlled cytokines and perforin in plasma samples collected at day 0, 2, 4 and 8 post-infection, using Milliplex Map NHP Cytokine Magnetic bead Panel assay. Each measurement of individual animals was done in duplicate, which was then averaged to constitute the individual value. For each cytokine, a linear regression was performed to evaluate if the slope of the fitted model was significantly different from 0. Normality of residuals were evaluated by a Shapiro-Wilk's test.

¤Normality of residuals was not assumed.

Supplementary Table 2. Summary of available experimental data on the ability of NiV W and its variants to accumulate in the nucleus, to bind to 14-3-3, to alter NF-kB p65 nucleo-cytoplasmic distribution and inhibit NF-κB -mediated signalling

Protein variant & source	NiV W Intracellular distribution	14-3-3		p65 (activated by IL1β/TNFα)			NF-κB Luc activated by IL1β/TNFα	NF-κB activation of transcription by IL1β/TNFα	Comments
		Binds to W	Distribution	Binding to W	Distribution	S536^{P}			
Empty vector			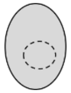		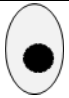	++	++	++	
W	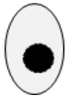	++	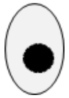	-	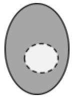	-	-	-	
P-NTD	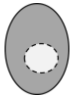	-	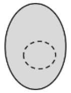	(-)[3]	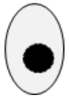	++	++	++	W-NTD is dispensable
W-CTD	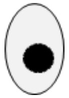	++	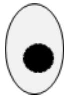	(-)[3]	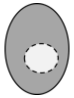	-	-	-	W-CTD is required
W-NLS^{0} [1]	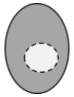	(+)[4]	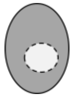	(-)[3]	nt[7]	nt	++	nt	Nuclear import of NiV W is required for NF-κB inhibition
W-NES^{0} [2]	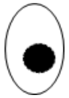	(+)[5]	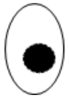	(-)[3]	nt	nt	-	nt	NES is dispensable for NiV W mediated inhibition of NF-κB signalling
W-NES^{0}NLS^{0}	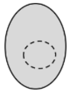	(+)[6]	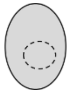	(-)[3]	nt	nt	++	nt	Loss of competition by importin α3 and α4 => binding of 14-3-3 can occur also in the cytosol
W-S199A	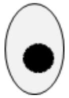	++	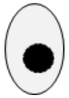	(-)[3]	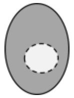	-	-	-	
W-S449A	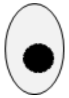	-	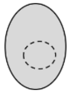	(-)[3]	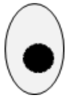	++	++	++	W-S449 mediates interaction with 14-3-3 and inhibition of NF-κB signalling
W-S199A-S449A	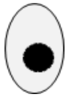	-	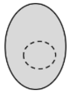	(-)[3]	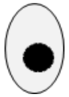	++	++	++	

[1] Alanine substitution: K439A, K440A, R442A
[2] Alanine substitution: L174A, L186A
[3] Inferred from lack of binding of wt W
[4] Inferred from W-CTD (lacking NTD in which NES is located)
[5] Data from Edwards *et al* 2020[41]
[6] Inferred from W-CTD (lacking NTD in which NES is located) and from NLS^{0} data in Edwards *et al* 2020[41]
[7]nt: not tested.
