# Supplementary material for: Nipah virus W protein harnesses nuclear 14-3-3 to inhibit NF-κB-induced proinflammatory response
Source: Commun Biol. 2021 Nov 16;4:1292. doi: 10.1038/s42003-021-02797-5 (PMC8595879; doi:10.1038/s42003-021-02797-5)
Supplement: Supplementary file 3 — Description of Additional Supplementary Files [file 42003_2021_2797_MOESM3_ESM.pdf]

Description of Additional Supplementary Files

File name: Supplementary Data 1.
Description: Source data.
