# Supplementary material for: Nipah virus W protein harnesses nuclear 14-3-3 to inhibit NF-κB-induced proinflammatory response
Source: Commun Biol. 2021 Nov 16;4:1292. doi: 10.1038/s42003-021-02797-5 (PMC8595879; doi:10.1038/s42003-021-02797-5)
Supplement: Supplementary file 5 — Reporting Summary [file 42003_2021_2797_MOESM5_ESM.pdf]

nature research

Corresponding author(s): Branka Horvat

Last updated by author(s): Sep 21, 2021

Reporting Summary

Nature Research wishes to improve the reproducibility of the work that we publish. This form provides structure for consistency and transparency in reporting. For further information on Nature Research policies, see our Editorial Policies and the Editorial Policy Checklist.

Statistics

For all statistical analyses, confirm that the following items are present in the figure legend, table legend, main text, or Methods section.

n/a	Confirmed	
☐	☒	The exact sample size (*n*) for each experimental group/condition, given as a discrete number and unit of measurement
☐	☒	A statement on whether measurements were taken from distinct samples or whether the same sample was measured repeatedly
☐	☒	The statistical test(s) used AND whether they are one- or two-sided<br>*Only common tests should be described solely by name; describe more complex techniques in the Methods section.*
☒	☐	A description of all covariates tested
☐	☒	A description of any assumptions or corrections, such as tests of normality and adjustment for multiple comparisons
☐	☒	A full description of the statistical parameters including central tendency (e.g. means) or other basic estimates (e.g. regression coefficient) AND variation (e.g. standard deviation) or associated estimates of uncertainty (e.g. confidence intervals)
☒	☐	For null hypothesis testing, the test statistic (e.g. *F*, *t*, *r*) with confidence intervals, effect sizes, degrees of freedom and *P* value noted<br>*Give P values as exact values whenever suitable.*
☒	☐	For Bayesian analysis, information on the choice of priors and Markov chain Monte Carlo settings
☒	☐	For hierarchical and complex designs, identification of the appropriate level for tests and full reporting of outcomes
☒	☐	Estimates of effect sizes (e.g. Cohen's *d*, Pearson's *r*), indicating how they were calculated

Our web collection on statistics for biologists contains articles on many of the points above.

Software and code

Policy information about availability of computer code

Data collection

LTCS SP5 LeicaTM Mycrosystems https://www.leica-microsystems.com/fr/produits/microscopes-confocaux/informations-detaillees/product/show/Products/leica-tcs-sp5/
LTQ Velos Mass Spectrometer Thermo Fisher Scientific
nanoLC Ultimate 3000 Thermo Fisher Scientific
LSM800 Zeiss
ImageStreamX MarkII Merck Millipore https://www.luminexcorp.com
StepOnePlus Real-Time PCR System Applied Biosystems and StepOnePlus v2.3 Thermo Fisher Scientific https://www.thermofisher
Magpic luminex Merck, Millipore
Infinite 200 Pro Lifesciences, TecanTM https://lifesciences.tecan.com/plate_readers/infinite_200_pro
Mithras LB940 BertholdTM https://www.berthold.com/en/bioanalytic/products/microplate-readers/mithras-lb940/
VersaDocTM Imaging System Bio-RadTM http://www.bio-rad.com/webroot/web/pdf/lsr/literature/Bulletin_5609.pdf
14-3-3-Pred: methods to predict 14-3-3-binding phosphopeptideshttp://www.compbio.dundee.ac.uk/1433pred

Data analysis

GraphPad Prism 8.3.0 GraphPad Software Inc. https://www.graphpad.com/scientific-software/prism
IDEAS® Image analysis Merck Millipore https://www.luminexcorp.com
ImageJ 1.52p FiJi package Image J https://imagej.net/Fiji
Proteome DiscovererTM v1.4 Thermo Fisher Scientific https://www.thermofisher.com/
Analysis of RT-qPCR results was done using the 2ΔΔCT model (Pfaffl M.W. 2001, PMID: 11328886), according to the MIQE guideline (Bustin SA et al, 2009, PMID: 19246619).

For manuscripts utilizing custom algorithms or software that are central to the research but not yet described in published literature, software must be made available to editors and reviewers. We strongly encourage code deposition in a community repository (e.g. GitHub). See the Nature Research guidelines for submitting code & software for further information.

Data

Policy information about availability of data

All manuscripts must include a data availability statement. This statement should provide the following information, where applicable:

- Accession codes, unique identifiers, or web links for publicly available datasets
- A list of figures that have associated raw data
- A description of any restrictions on data availability

The authors declare that the data supporting the findings of this study is available from the corresponding author upon request. All relevant data including the numerical and statistical source data that underlie the graphs in figures, are provided with the paper and extended data.

Field-specific reporting

Please select the one below that is the best fit for your research. If you are not sure, read the appropriate sections before making your selection.

☒ Life sciences ☐ Behavioural & social sciences ☐ Ecological, evolutionary & environmental sciences

For a reference copy of the document with all sections, see nature.com/documents/nr-reporting-summary-flat.pdf

Life sciences study design

All studies must disclose on these points even when the disclosure is negative.

Sample size	Sample size calculations were not performed. All experiments were done with replicates and successfully reporduced at least 2 times to demonstrate the magnitude and consistency of measurable differences.
Data exclusions	Data were not excluded.
Replication	All data reported in this study was reproduced in at least two independent biological replicates, as well as (when possible) via alternative independent methods.
Randomization	Samples were not randomised.
Blinding	Blinding was performed for the immunofluorescence analysis.

Reporting for specific materials, systems and methods

We require information from authors about some types of materials, experimental systems and methods used in many studies. Here, indicate whether each material, system or method listed is relevant to your study. If you are not sure if a list item applies to your research, read the appropriate section before selecting a response.

Materials & experimental systems

n/a	Involved in the study	
☐	☒	Antibodies
☐	☒	Eukaryotic cell lines
☒	☐	Palaeontology and archaeology
☐	☒	Animals and other organisms
☒	☐	Human research participants
☒	☐	Clinical data
☒	☐	Dual use research of concern

Methods

n/a	Involved in the study	
☒	☐	ChIP-seq
☒	☐	Flow cytometry
☒	☐	MRI-based neuroimaging

Antibodies

Antibodies used

Alexa 488 conjugated anti-rabbit InvitrogenTM Cat# A11034
Alexa 555 conjugated anti-mouse InvitrogenTM Cat# A31570
Alexa 647 conjugated streptavidin InvitrogenTM Cat# S21374
Anti-Flag antibody-coated magnetic beads SigmaTM Cat# M8823
Horseradish peroxydase conjugated anti-mouse IgG antibody PromegaTM Cat# W4021
Horseradish peroxydase conjugated anti-rabbit IgG antibody PromegaTM Cat# W4011
Mouse anti-Flag antibody SigmaTM Cat# F1804
Mouse anti-HA antibody SigmaTM Cat# H3663
Mouse anti-14-3-3 pan antibody Santa cruz Cat# sc- 1657
Mouse anti-GAPDH antibody ChemiconTM Cat# MAB374

	Rabbit anti-Flag Cell Signaling Cat#2368S<br>Rabbit anti-NF-kB p65 antibody Santa Cruz Cat# sc-372<br>Rabbit anti-14-3-3 pan antibody Merck milliporeTM Cat# AB9748-I<br>Rabbit anti-14-3-3 pan antibody Cell SignalingTM Cat# 8312<br>Rabbit anti-NF-kB p65 antibody Cell Signaling Cat# 8242S<br>Rabbit anti-NF-kB p65-P antibody Cell Signaling Cat# 3033S<br>Rabbit anti-NiV N antibody Valbex<br>Rabbit anti-NiV P/V/W-NTD (polyclonal raised against amino acids 1 to 407 of P/V/W)<br>Rabbit anti-NiV WΔCTD antibody (raised against the C-terminal portion of the W protein (amino acids 408 to 450) GeneScriptTM N/A<br>Rabbit NiV WΔCTD biotinylated antibody GeneScriptTM
Validation	All commercialy available antibodies were validated by the manufacturer and the data is available in their corresponding websites.<br>Rabbit anti-N antibody was walidated in our previous publication: Dhondt et al, 2012 PMID: 23089589.<br>Rabbit anti-NiV P/V/W-NTD was validated in the publcaiton Kulkarni et al, 2009, PMID: 19211754<br>Rabbit anti-NiV WΔCTD was initaly validated in ELISA assay using the correponding peptide and further validated in our lab, using sections from paraffine embeded organs of hamstres and monkeys infected with Nipah virus.

Eukaryotic cell lines

Policy information about cell lines

Cell line source(s)	Human carcinoma HeLa (Cat# ATCC CCL-2) was obtained form ATCC. HEK293T cells stably transduced with a NF-κB_luc reporter gene was provided by Dr C. Pique (Inserm, Paris) and HPMEC cells (human pulmonary microvascular endothelial cells) were provided by Dr. V. Krump-Kalinkova.
Authentication	The cell lines used were not authenticated. Cell lines originating from ATCC were extensively validated by ATCC. HEK293T and HPMEC lines were described in detail in references provided in the manuscript.
Mycoplasma contamination	All cell lines were tested for mycopasma on monthly bases and found to be uncontaminated.
Commonly misidentified lines<br>(See ICLAC register)	No commonly misidentified lines were used.

Animals and other organisms

Policy information about studies involving animals; ARRIVE guidelines recommended for reporting animal research

Laboratory animals	Three healthy 3-years old femalle African green monkeys from Saint Kitts were used in the study.
Wild animals	No wild anaimals were used in this study
Field-collected samples	No field-collected samples were used in this study.
Ethics oversight	Animals were handled in strict accordance with good animal practice as defined by the French national charter on the ethics of animal experimentation and all efforts were made to minimize suffering. Animal work was approved by the Regional ethical committee and French Ministry of High Education and Research and experiments were performed in the INSERM Jean Mérieux BSL-4 laboratory in Lyon, France (French Animal regulation committee N° D69 387 05 02).

Note that full information on the approval of the study protocol must also be provided in the manuscript.
